# Renewable and Recyclable Polymeric Materials for Food Packaging: A New Open Special Issue in *Materials*

**DOI:** 10.3390/ma15175845

**Published:** 2022-08-24

**Authors:** Shima Jafarzadeh, Masoumeh Zargar, Mehrdad Forough

**Affiliations:** 1 School of Engineering, Edith Cowan University, Joondalup, WA 6027, Australia; 2 Department of Chemistry, Middle East Technical University, 06800 Ankara, Turkey

“Renewable and Recyclable Polymeric Materials for Food Packaging” is a new open Special Issue of *Materials* that will publish original and review papers on new scientific and applied research, and the articles it contains will make a contribution to the discovery and understanding of biodegradable and recyclable materials, their functional properties, characterization and applications.

Plastic is a common material used in food packaging. However, as plastics are petroleum-based, they take centuries to degrade. In fact, fewer than 12% of plastics are recycled, and an estimated 95% are discarded after one use. Thus, non-biodegradability and disposal problems are the major challenges associated with synthetic plastic packaging. A vast amount of plastic is disposed of annually by being left in landfills, incinerated, or dumped in the oceans worldwide, as this is the cheapest and fastest way to dispose of generated waste [1]. It is predicted that plastic production and incineration will release more than 850 million tonnes of greenhouse gases this year, a figure that is estimated to reach to 2.8 billion tonnes by 2050 [2]. The significant damage that plastics cause to the environment means that research into the environmental hazards of plastics and the development of strategies to remove and control plastics from the environment is a priority. Renewable and recyclable polymeric materials are the best option to solve the problem of synthetic plastic waste.

While researchers have been working on developing biodegradable packaging in recent years, consumers want food packaging materials that are biodegradable, recyclable, environmentally friendly, and capable of extending the shelf life of food. In this context, bioactive packaging has emerged as a way to produce sustainable, efficient, and versatile packaging.

An advanced polymer system containing modifying agents is characterized as a combination of materials with improved properties (e.g., mechanical, thermal, antimicrobial, and barrier properties, conductivity, and biodegradability) or functions (e.g., the ability to release additives) that are used for a variety of industrial applications. Blending environmentally friendly biopolymers with renewable natural sources, synthetic polymers, nanofillers, natural anti-browning agents, nutrients, colorants, natural antioxidants, and antimicrobial compounds can result in a unique combination of properties that enhance products’ quality, safety, and shelf life while reducing bacteria growth. Therefore, the development of tailored polymer systems will allow us to meet technological challenges while addressing global sustainability issues [3].

The research interests of the section *Renewable and Recyclable Polymeric Materials* include the development of biodegradable smart/intelligent packaging, characterizations, and the incorporation of natural functional ingredients, antioxidants, antimicrobial compounds, and eco-friendly nanomaterials to enhance the properties of biopolymers.

As Guest Editor, I am delighted to invite contributions on this topic in the form of original research articles or reviews.
